# Chemical Composition of *Cynanchum auriculatum* Royle Ex Wight and Its Potential Role in Ameliorating Colitis

**DOI:** 10.1002/fsn3.4764

**Published:** 2025-01-19

**Authors:** Sichen Li, Yuning Sun, Huihui Peng, Ruiqiang You, Fuqing Bai, Dan Chen, Mohamed Abdin, Chuanyi Peng, Xiang Li, Huimei Cai, Guijie Chen

**Affiliations:** ^1^ State Key Laboratory of Tea Plant Biology and Utilization, School of Tea & Food Science and Technology Anhui Agricultural University Hefei Anhui P.R. China; ^2^ Joint Research Center for Food Nutrition and Health of IHM Anhui Agricultural University Hefei Anhui P.R. China; ^3^ School of Marine and Biological Engineering Yancheng Teachers' University Yancheng China; ^4^ College of Food Science and Engineering Yangzhou University Yangzhou Jiangsu China; ^5^ Agricultural Research Center Food Technology Research Institute Giza Egypt

**Keywords:** chemical composition, colitis, *Cynanchum auriculatum*, gut microbiota, short‐chain fatty acids

## Abstract

*Cynanchum auriculatum* Royle ex Wight, commonly known as “Baishouwu,” has been traditionally used in China for its medicinal and dietary benefits. Despite its long history of use, the potential therapeutic effects of *C. auriculatum* in the treatment of colitis have not been fully investigated. This study aims to evaluate the effects of the water extract of *C. auriculatum* root on colitis and elucidate its potential mechanisms of action. The water extract of *C. auriculatum* root (CW) was prepared and characterized using UPLC‐Q‐TOF‐MS, identifying thirty‐two distinct compounds, including saponins, organic acids, fatty acid derivatives, and alkaloids. The therapeutic efficacy of CW was assessed in a colitis mouse model. CW significantly alleviated colitis symptoms, evidenced by increased colon length, reduced disease activity indices, and decreased colon tissue damage. CW reduced colonic inflammatory cytokine production and enhanced the expression of tight junction proteins, including claudin‐1, occludin, and ZO‐1, thereby strengthening intestinal barrier integrity. Additionally, CW modulated the gut microbiota by increasing microbial diversity, promoting beneficial *Lactobacillus* growth, reducing pathogenic *Pseudomonas* levels, and enhancing short‐chain fatty acid production. The results suggest that CW exhibits significant therapeutic potential in the management of colitis by attenuating inflammation, restoring gut barrier function, and modulating the gut microbiota. These findings provide a basis for further exploration of *C. auriculatum* as a functional food for prevention and treatment of colitis.

## Graphical abstract

In this study, the water extract of *Cynanchum auriculatum* Royle ex Wight root (CW) was prepared and its chemical composition was characterized using UPLC‐Q‐TOF‐MS. CW significantly alleviated DSS‐induced colitis in mice, as demonstrated by increased colon length, reduced disease activity index scores, and decreased histological damage in colon tissues. This study provides the first evidence of a complex interplay between CW and colitis mitigation, which is mediated by elevated levels of short‐chain fatty acids and the upregulation of tight junction protein expression.

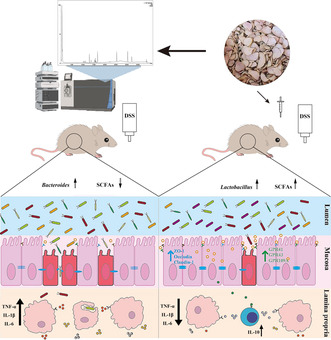

## 1 Introduction

Inflammatory bowel disease (IBD) represents a group of persistent inflammatory disorders that affect the gastrointestinal tract and substantially impact the lives of millions globally. This category encompasses primarily ulcerative colitis (UC) and Crohn's disease (CD). Symptoms of IBD, including abdominal pain, watery diarrhea, rectal bleeding, and weight loss, severely impair the quality of life for patients (Flynn and Eisenstein 2019). Recent statistics indicate a marked increase in IBD prevalence, with over 1.5 million affected individuals in Europe and 2 million in North America (Neurath 2019), particularly affecting younger populations. Furthermore, IBD also elevates the risk of developing colorectal cancer (Shah and Itzkowitz 2022). The complex etiology of IBD involves immune dysregulation, genetic predispositions, lifestyle factors, and microbial imbalances within the gut. Despite the availability of treatments such as aminosalicylates, corticosteroids, and immunosuppressants, these often result in side effects and provide only temporary relief (Liu, Di, and Xu 2023). Consequently, there is a critical need to develop new, long‐lasting treatments that minimize adverse effects.

The human gastrointestinal tract is home to approximately 100 trillion microbes, which play a crucial role in regulating a range of physiological and metabolic functions essential for gastrointestinal and overall digestive health (Muramatsu and Winter 2024). The development of IBD is closely associated with dysbiosis of the gut microbiota, a connection that is particularly pronounced in UC (Franzosa et al. 2019). In studies using a dextran sulfate sodium (DSS)‐induced colitis model in mice, normal mice develop colitis while germ‐free mice show little or no intestinal inflammation (Forster et al. 2022), highlighting the significant role of the gut microbiota in intestinal inflammation. The gut microbiota in UC patients is characterized by reduced bacterial diversity, altered composition, and an increase in pathogenic microbes, along with a concurrent decrease in probiotics (Herrera‐deGuise et al. 2023). This ecological imbalance could compromise the intestinal barrier function, leading to abnormal immune responses, increased intestinal permeability, and enhanced inflammation. Short‐chain fatty acids (SCFAs), including acetate, propionate, and butyrate, are essential in regulating immune cell activity, energy metabolism, and sustaining the equilibrium of the intestinal environment (Luo et al. 2022; Yao et al. 2022). The recent works showed a reduction in key metabolic products like SCFAs in IBD patients is associated with these microbial changes (Deleu et al. 2021). As a result, adjusting the gut microbiota through the use of probiotics has been recognized as a potentially effective method for treating IBD.

Patients with UC exhibit elevated levels of several pro‐inflammatory factors within their intestinal mucosa, including tumor necrosis factor‐alpha (TNF‐α), interleukin‐6 (IL‐6), and IL‐1β (Bergemalm et al. 2021). The stability of the gastrointestinal environment is critically dependent on the intricate interplay between the gut microbiota, intestinal epithelial cells, and the resident immune cells. In cases of ulcerative colitis (UC), the structural integrity of the intestinal barrier is largely reliant on the functionality of tight junction (TJ) proteins including claudins, occludin, and zonula occludens‐1 (ZO‐1). Dysfunction in these proteins leads to diminished barrier protection and increased intestinal permeability, which facilitates the infiltration of pathogens and antigens, thereby initiating an immune response (Zeisel, Dhawan, and Baumert 2019). Mucin‐2 (MUC2) plays a pivotal role in gut protection as a major constituent of the protective mucus secreted by goblet cells, essential for maintaining intestinal integrity. In MUC2 knockout mice, the colonic epithelial resistance to bacterial invasion is significantly weakened, indicating the pivotal role of MUC2 in maintaining intestinal integrity (Yao et al. 2021). There is increasing evidence that modulating the expression of TJ proteins to restore epithelial barrier integrity, thereby mitigating and preventing inflammation, presents a promising treatment avenue worth further exploration. Targeting the expression and functionality of tight junction proteins, along with MUC2, represents a promising new therapeutic strategy to enhance intestinal barrier integrity in inflammatory bowel disease (IBD). This approach underscores the barrier's critical role in IBD pathology and introduces novel opportunities for intervention.

*Cynanchum auriculatum* Royle ex Wight is widely distributed across China. Historically, the root of *C. auriculatum* Royle ex Wight, commonly known as “Baishouwu,” has been celebrated as a tonic herbal medicine with thermogenic properties, utilized both in clinical settings and as a health food since ancient times (Chai et al. 2018). The various compounds, including stilbene glycosides, phenylketonuria, and C21 steroidal glycosides, have been identified from Baishouwu (Gan et al. 2008; Teng et al. 2011). Research in pharmacology has revealed that Baishouwu possesses a range of biological effects, including anti‐tumor properties, immune enhancement, and antioxidant capabilities (Chai et al. 2018; Ding et al. 2019). However, its precise impact on inflammatory bowel disease (IBD) remains unclear.

In this investigation, the water extract of *C. auriculatum* Royle ex Wight root (CW) was prepared and its components identified through UPLC‐Q‐TOF‐MS. The therapeutic effects on colitis were assessed using a DSS‐induced colitis model in mice. Further, 16S rRNA sequencing technology helped explore the underlying mechanisms. It is noteworthy that this research is pioneering in showing CW's capacity to alleviate colitis by modulating gut microbiota and boosting SCFAs production. These findings offer fresh perspectives on Baishouwu's utility in colitis treatment and underscore the significance of microbial interactions.

## 2 Materials and Methods

### 2.1 Materials and Reagents

The root of *C. auriculatum* Royle ex Wight was sourced from Binhai County, Jiangsu Province. DSS, with a molecular weight range of 36,000–50,000, was purchased from MP Biomedicals (Santa Ana, California, Catalogue No.: 160110). Serum TNF‐α, IL‐1β, and IL‐6 levels were measured using ELISA kits from Nanjing Jiancheng Bioengineering Institute (Jiangsu, China). Standards for acetic, propionic, n/i‐butanolic, and n/i‐pentanoic acids were procured from Aladdin Chemical Reagent Co. Ltd. (Shanghai, China), while 2‐ethylbutanoic acid was supplied by Shanghai Yuan Ye Biotechnology Co. Ltd. All other chemicals, of analytical grade, were obtained commercially.

### 2.2 Preparation of CW

Given that Baishouwu is typically consumed after boiling in water, our study focused on its aqueous extracts. The extraction method for CW was based on Chen et al. with minor modifications (Chen et al. 2018). Briefly, 50 g of *C. auriculatum* root powder was extracted in distilled water at a 1:20 ratio and heated at 100°C for one hour. This extraction process was repeated three times, with filtration performed after each cycle. The collected filtrates were then combined, centrifuged, concentrated under reduced pressure, and freeze‐dried to produce the CW samples.

### 2.3 Chemical Analysis of CW

The chemical composition of CW was analyzed using an ACQUITY Xevo G2‐XS Q‐TOF system (Waters, Milford, MA, USA), integrated with UPLC‐Q‐TOF‐MS. Chromatographic separation was carried out on a Waters ACQUITY UPLC BEH C18 column (2.1 mm × 50 mm, 1.7 μm), maintained at a column temperature of 40°C and a flow rate of 0.4 mL/min. The mobile phase included 0.1% formic acid in water (A) and 0.1% formic acid in acetonitrile (B). The gradient elution program was set as follows: 0–0.2 min, 5% B; 0.2–2 min, 5%–8% B; 2–9 min, 8%–13% B; 9–16 min, 13%–20% B; 16–16.5 min, 20%–21% B; 16.5–22 min, 21%–30% B; 22–23 min, 30%–35% B; 23–28 min, 35%–42% B; 28–33 min, 42%–60% B; 33–35 min, 60%–98% B; 35–38 min, 98% B; 38–40 min, 2% B. Q‐TOF/MS analysis was performed using electrospray ionization (ESI) in both positive and negative ion modes for a full‐scan analysis. The source parameters were optimized to 2 kV in positive ion mode and 1.5 kV in negative ion mode, with an ion source temperature of 100°C and a desolvation temperature of 400°C. Nitrogen served as the nebulizing gas at a flow rate of 800 L/h. The mass acquisition range spanned from 50 to 1200 m/z, with collision energies varying from 20 to 40 V using high‐purity argon as the collision gas. Data acquisition and management were conducted using UNIFI software version 1.9.4.

### 2.4 Animals and Experimental Design

All animals experiment was performed in strict compliance with the guidelines stipulated by the National Research Council's Guide for the Care and Use of Laboratory Animals. The diets for the animals were supplied by Jiangsu Xietong Pharmaceutical Bio‐engineering Co. Ltd. (Jiangsu, China), with the specific dietary compositions detailed in Table S1. Male Specific Pathogen‐Free (SPF) C57BL/6J mice, aged 8 weeks, were obtained from Jiangsu Huachuang Xinuo Pharmaceutical Technology Co. Ltd., and maintained under stringent SPF conditions at the Anhui Agricultural University animal facility. Following a 7‐day acclimatization period, the mice were randomly allocated into three groups, each consisting of eight individuals: a control group (Control), which received 200 μL of sterile water daily via gavage; a model group (DSS), which followed the same gavage regimen, with 1.5% (w/v) DSS supplemented in their drinking water from day 14 for 11 consecutive days (Li et al. 2024), and thereafter, sterile water for another 7 days; and an intervention group (CW), treated identically to the model group but with sterile water replaced by 200 mg/kg day of CW. The variables such as dietary intake, water consumption, body weight, fecal occult blood presence, and any indications of bleeding for each group were meticulously recorded. Disease Activity Index (DAI) scores were calculated based on blinded evaluations according to the previous method (Xie et al. 2023) as shown in Table S2. On the 32nd day of the study, all mice were humanely euthanized, and data regarding spleen weight and colon length were collected. In addition, fresh fecal samples were gathered three days prior to the experiment for subsequent biochemical and histological analyses. The entire experimental protocol was sanctioned by the Animal Ethics Committee of Anhui Agricultural University (Approval No. AHAU2023028).

### 2.5 Histological and Immunofluorescence Analysis

Fresh colon tissues were processed as follows: fixation in 4% paraformaldehyde for 24 h, followed by dehydration in a graded ethanol series. After embedding in paraffin, the tissues were sectioned into 5 μm slices. These sections were stained with Hematoxylin and Eosin (H&E) to assess general morphology and with Alcian Blue‐Periodic Acid Schiff (AB‐PAS) for mucin detection. The stained sections were examined blindly using an optical microscope, with high‐resolution scanning images captured for detailed morphological analysis. Histological assessment of the H&E sections adhered to the criteria in Table S3, while goblet cell quantification in AB‐PAS‐stained tissues used ImageJ software.

During the immunofluorescence analysis of colon sections, antigen retrieval was performed in EDTA buffer with heating to expose the target antigens. The sections were then washed with PBS (pH 7.4) and incubated overnight at 4°C with primary antibodies (Anti‐ZO‐1, Anti‐Occludin, and Anti‐Claudin‐1) supplied by Abcam, Cambridge, UK. Subsequent steps included washing the sections three times in PBS, each for five minutes, and incubating them with FITC‐labeled secondary antibodies in darkness for one hour. After three more PBS washes, nuclear counter staining was done using 4′,6‐diamidino‐2‐phenylindole (DAPI) at 25°C for 10 min, followed by a final set of three PBS washes. Fluorescence microscopy images were captured and pixel intensity was analyzed using ImageJ software, ensuring unbiased results through a double‐blind protocol.

### 2.6 Serum Biochemical Analysis and Colon mRNA Analysis

In this study, serum levels of TNF‐α, IL‐1β, and IL‐6 were measured using commercial ELISA kits as per the manufacturer's guidelines. Total RNA was extracted from colonic homogenates with the FastPure Cell/Tissue Total RNA Isolation Kit (Vazyme Biotech Co. Ltd., China), and its purity and concentration were assessed using a NanoDrop 2000 spectrophotometer (Thermo Fisher Scientific, USA). The RNA was then converted into complementary DNA (cDNA) using PrimeScript RT Master Mix and amplified via SYBR Green Fast Mix on the QuantStudio 6 Flex Real‐Time PCR System (Thermo Fisher Scientific, USA). The relative mRNA expression of target genes was quantitatively analyzed using the 2‐ΔΔCt method, normalized against GAPDH. Primer sequences are provided in Table S4.

### 2.7 Quantification of SCFAs in Serum and Stool Samples

The concentrations of acetic, propionic, n/i‐butanoic acid, and n/i‐pentanoic acid were determined using a modified method (Xie et al. 2017). Samples were prepared and analyzed using an Agilent 7890 N Gas Chromatograph (GC) from Agilent Technologies. Fecal samples were diluted in distilled water (1:5), centrifuged at 12,000 *g* for 5 min, and the supernatant was collected. This was acidified with 0.2 M HCl containing 0.3 mg/mL of 2‐ethylbutyric acid (internal standard), filtered through a 0.22 μm membrane, and stored at 4°C. Serum samples were prepared by mixing 50 μL of serum with 200 μL of the internal standard, vortexing, centrifuging, and filtering through a 0.22 μm membrane before storage at 4°C. GC analysis was conducted on an HP‐Innowax capillary column (30 m × 0.25 mm × 0.25 μm) using nitrogen as the carrier gas at 19 mL/min, with specific flow rates for air, hydrogen, and make‐up nitrogen. The temperature program started at 100°C for 1 min, ramped to 180°C at 5°C/min, held for 16 min, and finally maintained at 180°C for 4 more minutes.

### 2.8 Analysis of the 16S rRNA Gene Sequencing

DNA was extracted from fecal samples using the FastDNA SPIN Kit for Soil (MP Biomedicals, Santa Ana, California), focusing on the V3 and V4 regions of the bacterial 16S rRNA gene. Amplification was performed via PCR with barcode‐specific primers, consisting of Forward Primer (Illumina adapter sequence 1 + CCTACGGGNGGCWGCAG) and Reverse Primer (Illumina adapter sequence 2 + GACTACHVGGGTATCTAATCC), targeting the aforementioned regions for precise amplification. The PCR products were sequenced on the Illumina NovaSeq 6000 platform by Genesky Biotechnologies Inc. (Shanghai, China), known for its high throughput and accuracy. Sequencing data analysis involved evaluating reads and identifying amplicon sequence variants (ASVs), enabling a detailed assessment of bacterial diversity and composition within the samples for microbial community studies.

### 2.9 Statistical Analysis

Statistical analysis was performed in SPSS, presenting results as mean ± standard deviation (SD). GraphPad Prism version 8.0 was utilized for data visualization. Group differences were evaluated by one‐way ANOVA with subsequent Tukey post hoc tests to identify specific differences. Significance levels were denoted as *p < 0.05, **p < 0.01, ***p < 0.001, indicating statistically significant differences. Alpha‐diversity and beta‐diversity analyses were processed in QIIME2, with further statistical analysis and visualization executed in R software, version 3.5.0.

## 3 Result

### 3.1 Chemical Constituents of CW

**FIGURE 1:**
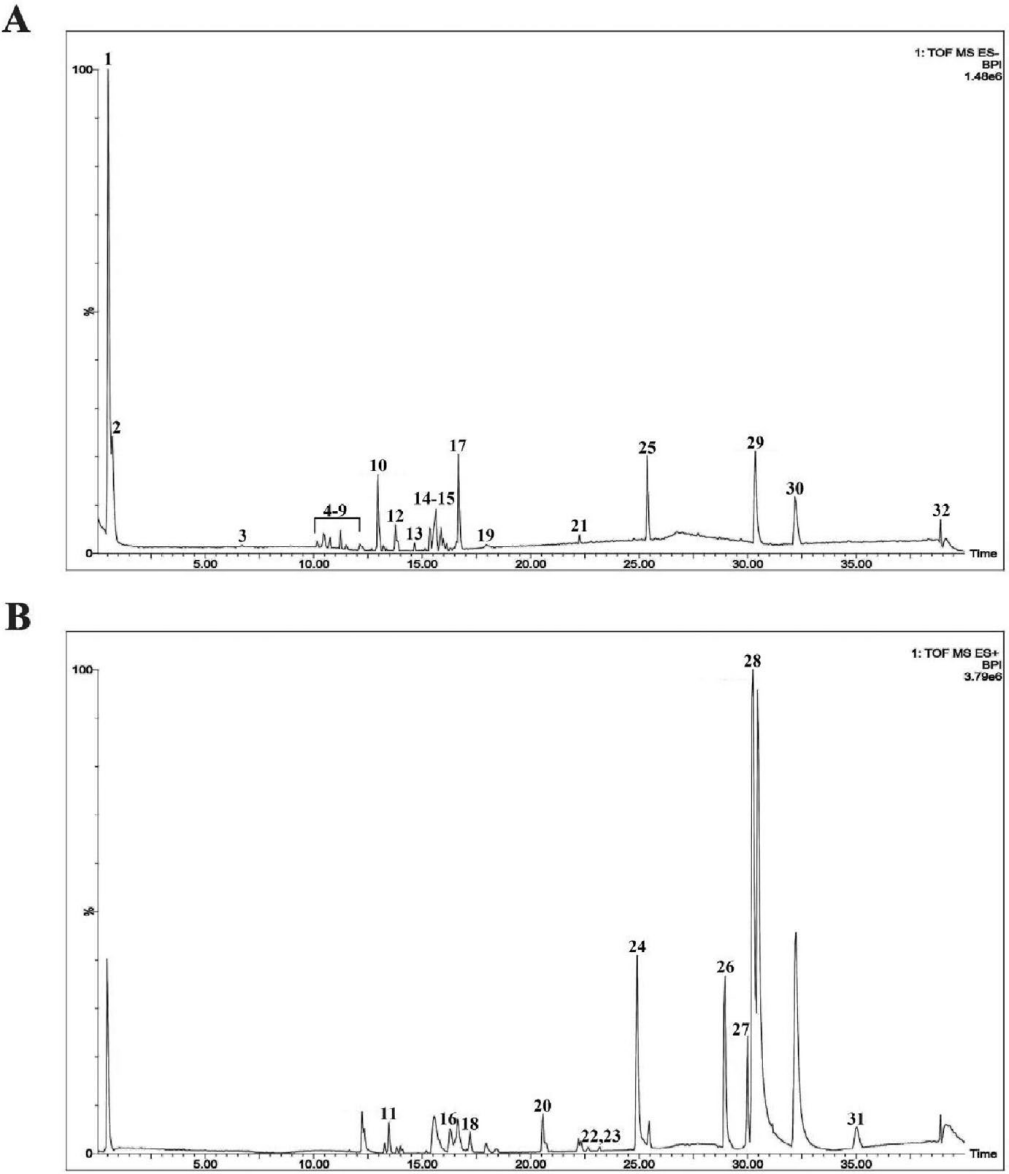
Chemical profiles of CW analyzed using UPLC‐Q‐TOF‐MS. Total ion chromatogram of CW in (A) negative and (B) positive models. 1: L‐Arginine, 2: Citric Acid, 3: Yesanchinoside B, 4: Esculentoside A, 5: Tianshic acid, 6: Isoacteoside, 7: Soyasaponin Bb, 8: 3‐O‐α‐L‐rhamnopyranosyl‐(1 → 2)‐α‐L‐arabinopyranosyl‐28‐O‐β‐D‐glucopyranosyl‐(1 → 6)‐β‐D‐glucopyranosyl oleanolate, 8: Buddleoside, 9: Yuzhizioside IV, 10: Palmitic acid, 11: Songoroside A, 12: Phytolaccagenin, 13: Coronaric acid, 14: Prosapogenin 5, 15: Lablaboside A, 16: Gandoeric acid H, 17: Raddeanoside R0, 18: Phytolaccagenin, 19: Ethyl linolenate, 20: Lobelanidine, 21: Nomilinic acid, 22: Glycerol monostearate, 23: Tribulosin, 24: Asterbatanoside D, 25: 3β‐Acetoxy‐atractylone, 26: Isoacteoside, 27: Cimicifuga Dahurica C, 28: Phytolaccagenin, 29: Platycoside D, 30: Lycopodium Alkaloids, 31: Asterbatanoside D.

In this work, UPLC‐Q‐TOF‐MS was utilized to characterize the metabolites in CW. The identification was enhanced by the use of UNIFI software, detailed fragmentation pathway analysis, and a thorough comparison with existing literature, and 32 distinct compounds including twelve saponins (Soyasaponin Bb, 3‐O‐α‐L‐rhamnopyranosyl‐(1 → 2)‐α‐L‐arabinopyranosyl‐28‐O‐β‐D‐glucopyranosyl‐(1 → 6)‐β‐D‐glucopyranosyl oleanolate, Buddleoside, Yesanchinoside B, Esculentoside A, Yuzhizioside IV, Songoroside A, Prosapogenin 5, Lablaboside A, Gandoeric acid H, Raddeanoside R0, and Tribulosin), four organic acids (Citric Acid, Tianshic acid, Coronaric acid, and Nomilinic acid), three fatty acid derivatives (Palmitic acid, Ethyl linolenate, and Glycerol monostearate), and two alkaloids (Lycopodium Alkaloids, and Lobelanidine) were identified. These findings are summarized in Figure 1 and Table S5.

### 3.2 CW Alleviated Clinical Symptoms in a Colitis Model

This study employed DSS to induce colonic epithelial damage in mice, simulating the clinical symptoms of UC (Wirtz et al. 2017). Key parameters such as body weight, DAI scores, colon length, and rectal bleeding were monitored to establish a colitis model. Over the initial 14 days of CW treatment, there were no significant changes in body weight percentage, water intake, or food intake among the mice compared to the control and DSS groups (Figure S1A–C). Notably, water and food intake in the DSS‐treated group decreased significantly compared to the control group, as shown in Figure S1D,E, but these parameters significantly recovered after cessation of DSS treatment, particularly in the CW‐treated group.

**FIGURE 2:**
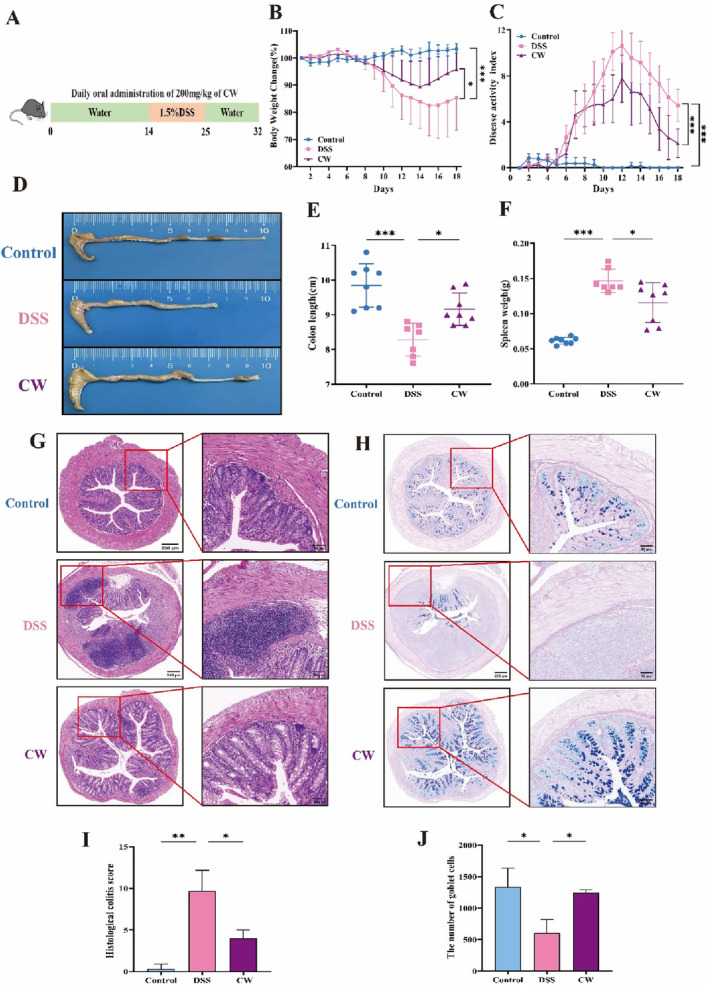
Ameliorating effect of CW on colonic damage. (A) Schematic overview of experimental design, (B) Body weight changes, (C) DAI, (D) Representative colonic tissue images from each group, (E) Colon length quantification, (F) Spleen weight, (G) H&E staining and (H) AB‐PAS staining of colon tissue, (I) Histopathologic scores, and (J) goblet cell count (*n* = 3 per group). Data are presented as mean ± SD (*n* = 7–8). Significance differences: *p < 0.05, **p < 0.01, ***p < 0.001.

Starting from day 6, the DSS group showed a significant reduction in body weight. In contrast, the CW treatment significantly countered this weight loss (Figure 2B). Additionally, CW notably decreased the DAI scores that DSS had elevated, suggesting its efficacy in easing colitis symptoms (Figure 2C). We assessed inflammation by measuring changes in colon length and spleen weight, finding that DSS significantly shortened the colon and increased the spleen weight. These effects were substantially mitigated by CW treatment (Figure 2D–F). Histopathological assessments showed that the control group had well‐preserved epithelial structures without abnormalities. In contrast, the DSS group displayed disorganized epithelium and extensive damage including disrupted crypt architecture and inflammation throughout the submucosal and mucosal layers (Figure 2G). Contrastingly, CW treatment significantly improved these colonic pathologies, repairing mucosal damage and reducing inflammatory cell infiltration, which resulted in lower histopathological scores than those seen in the DSS group (Figure 2I).

Goblet cells, crucial for mucin production and maintaining mucosal barrier integrity (Gustafsson and Johansson 2022), were abundant and well‐structured in the control group, as demonstrated in Figure 2H,J. In contrast, the DSS‐treated mice showed a marked decrease or absence of these cells, suggesting impaired mucin production. Notably, CW treatment effectively countered this reduction, preserving goblet cell integrity. Overall, CW significantly reduced the symptoms of DSS‐induced colitis, highlighting its potential as a therapeutic agent for maintaining mucosal health.

### 3.3 CW Mitigated DSS‐Induced Colonic Damage by Modulating Inflammatory Responses and Enhancing Tight Junction Protein Expression

**FIGURE 3:**
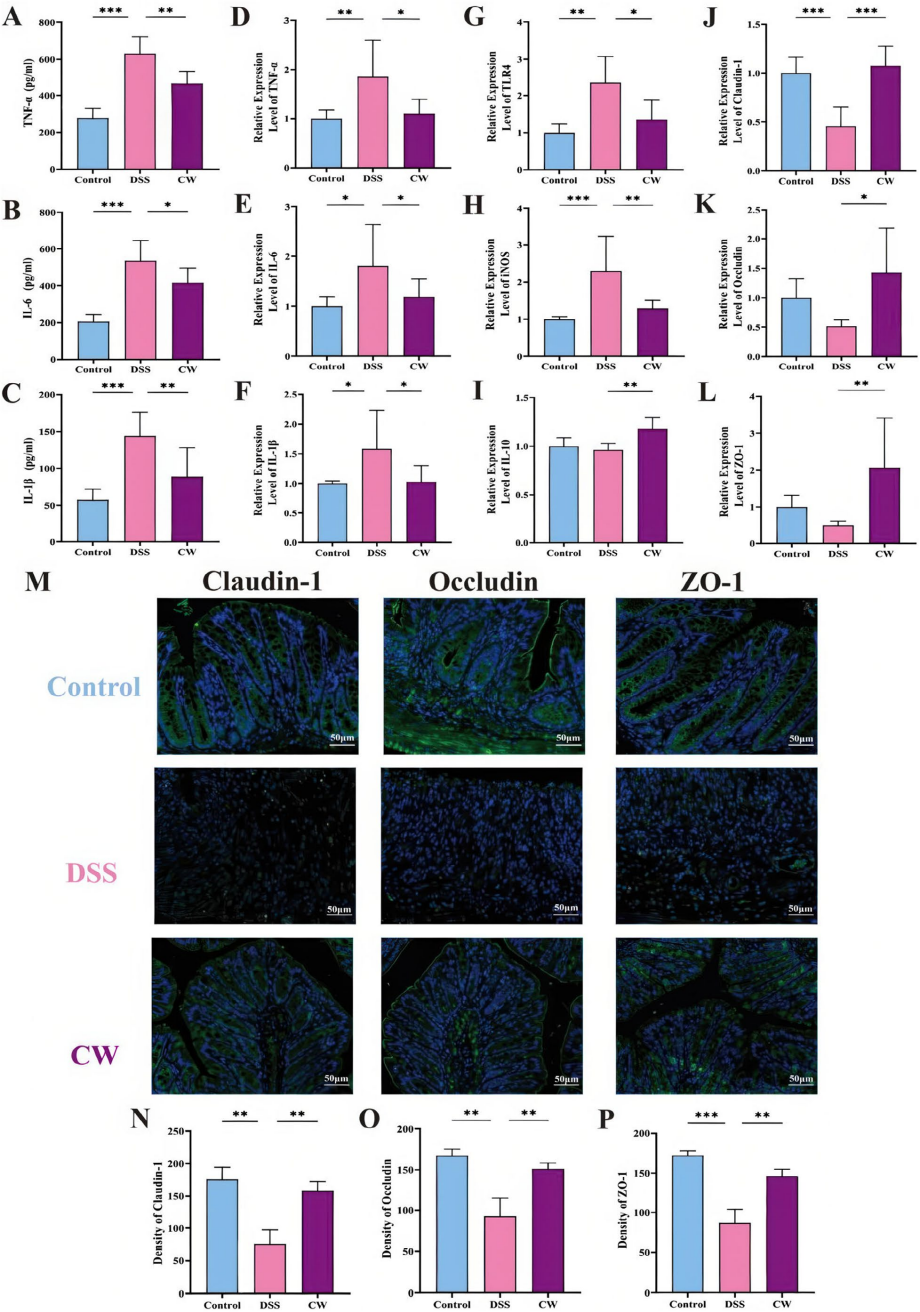
CW inhibited inflammatory cytokines and enhanced expression of tight junction (TJ) proteins. Analysis of serum cytokine levels of (A) TNF‐α, (B) IL‐1β, and (C) IL‐6, the mRNA levels of (D) TNF‐α, (E) IL‐1β, (F) IL‐6, (G) TLR4, (H) iNOS, (I) IL‐10, (J) ZO‐1, (K) Occludin, (L) Claudin‐1, (M) Representative immunofluorescence images of colonic sections stained for tight junction proteins; green indicates positive protein staining, and nuclei are counterstained in blue, Scale bar = 50 μm. (N–P) Quantitative analysis of protein density for ZO‐1, Occludin, and Claudin‐1 (*n* = 3 per group). All data are presented as mean ± SD (*n* = 7–8). Significance differences: *p < 0.05, **p < 0.01, ***p < 0.001.

Inflammatory cytokines play a critical role in IBD pathogenesis (Neurath 2024). To assess the effects of DSS and CW on inflammation, TNF‐α, IL‐1β, and IL‐6 serum concentrations were measured, revealing that DSS notably increased these levels compared to controls. Conversely, CW oral administration markedly lowered these concentrations (Figure 3A–C). Additionally, DSS elevated pro‐inflammatory cytokine mRNA levels in colonic tissues, while CW treatment not only reduced these levels but also enhanced IL‐10 expression, an anti‐inflammatory marker (Figure 3D–I). These results indicated that CW can mitigate DSS‐induced systemic inflammation and colitis at the cytokine level.

Furthermore, IBD pathogenesis often involves compromised intestinal epithelial barriers, primarily maintained by tight junction (TJ) complexes such as ZO‐1, occludin, and claudin‐1 (Zeisel, Dhawan, and Baumert 2019). DSS exposure decreased mRNA and protein levels of these critical proteins, suggesting increased intestinal permeability (Figure 3J–P). In contrast, CW supplementation led to an upregulation of these genes and a notable enhancement in their protein expression, underscoring CW's role in bolstering intestinal barrier function and maintaining intestinal health.

### 3.4 CW Promotes SCFAs Production and G Protein‐Coupled Receptors (GPCRs) Activation

**FIGURE 4:**
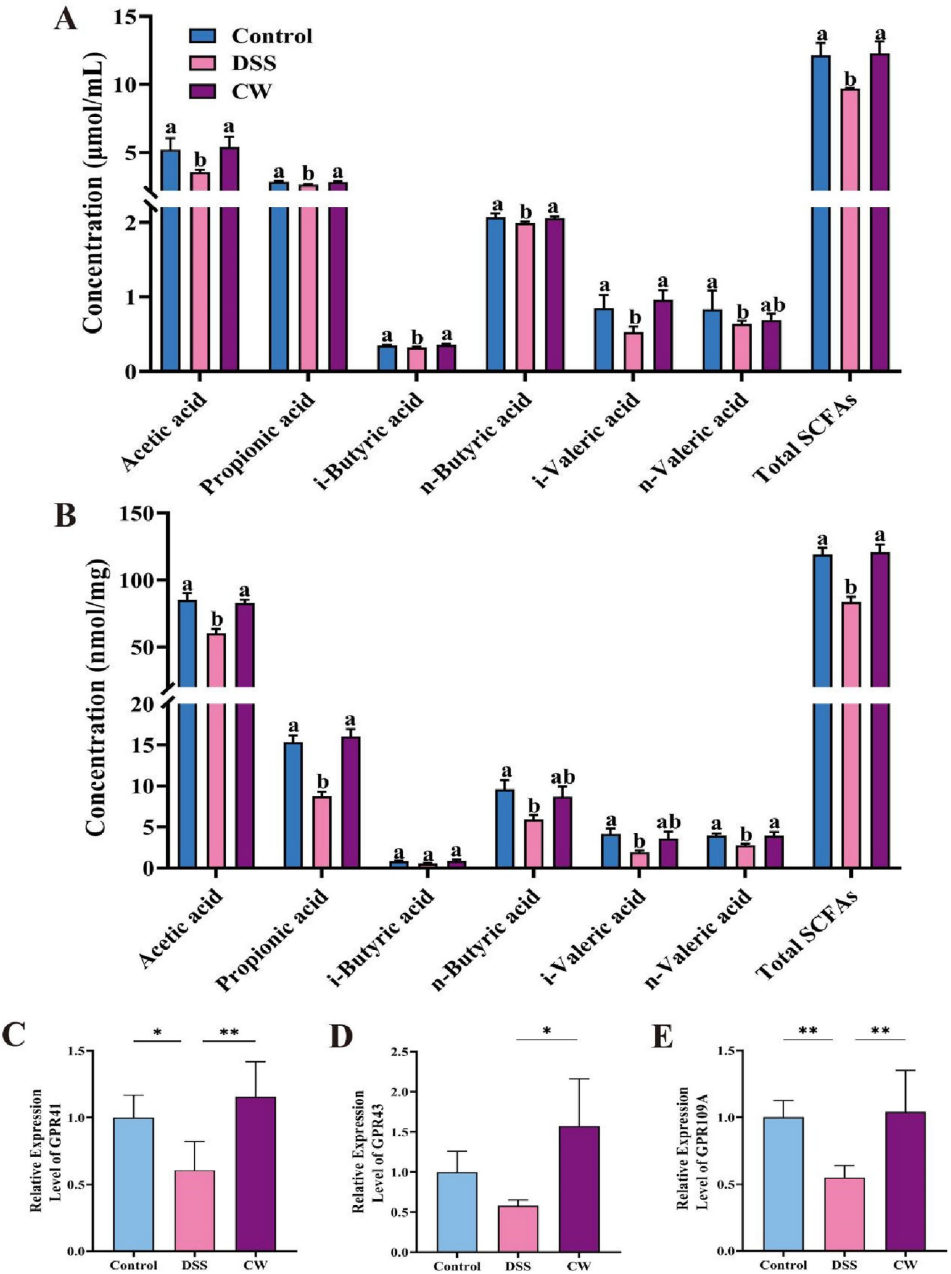
CW enhanced SCFAs levels in DSS‐induced colitis. The impact of CW on the concentrations of SCFAs in (A) serum and (B) colonic contents of mice, the mRNA expression levels of SCFAs receptors in the colon: (C) GPR41, (D) GPR43, and (E) GPR109A. All data are presented as mean ± SD (*n* = 6–8). Significance differences: *p < 0.05, **p < 0.01.

SCFAs, synthesized by gut microbiota, play a pivotal role in enhancing gut health and fortifying intestinal barrier function (He et al. 2024). Data presented in Figure 4A,B indicate that colitis, induced by DSS, led to reduced SCFAs levels in serum and colonic samples. Conversely, CW administration significantly elevated concentrations of acetate, propionate, isobutyrate, butyrate, and isovalerate in these samples, along with an overall increase in total SCFAs content.

Additionally, SCFAs act as signaling molecules by interacting with GPCRs like GPR41, GPR43, and GPR109A on cell surfaces, crucial for the modulation of anti‐inflammatory responses (Tan, Macia, and Mackay 2023). Findings from this study demonstrate that DSS exposure resulted in decreased GPR41 and GPR109A mRNA levels in colonic tissues, a trend that CW administration effectively countered, as depicted in Figure 4C–E. These results suggest that CW not only substantially boosts SCFAs production but also ameliorates inflammation through the modulation of GPCR‐mediated signaling pathways in the gut.

### 3.5 CW's Role in Counteracting DSS‐Induced Colitis Through Gut Microbiota Modulation

**FIGURE 5:**
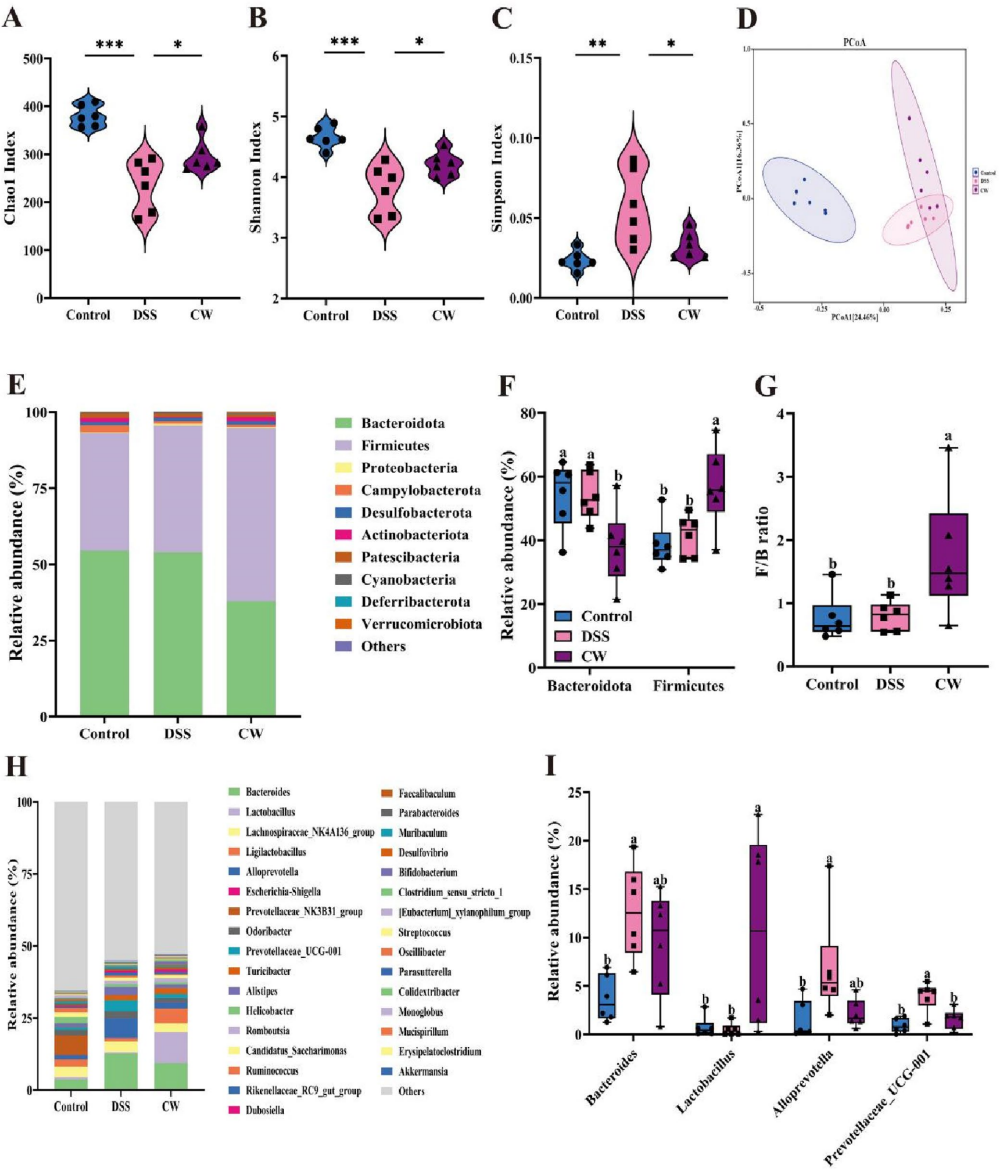
CW treatment modulated gut microbiota structure in DSS‐induced colitis. α‐Diversity indices including (A) Chao1, (B) Shannon, and (C) Simpson; (D) β‐diversity analyzed via principal coordinates analysis (PCoA). The composition of gut microbiota at the phylum level (E), the relative abundances of Firmicutes and Bacteroidetes (F) and the Firmicutes/Bacteroidetes ratio (F/B) (G). The genus‐level taxonomic profiling (H), with significantly altered taxa at the genus level (I). All data are presented as mean ± SD (*n* = 6). Significance differences: *p < 0.05, **p < 0.01, ***p < 0.001.

Disruptions in the gut microbiota can lead to abnormal immune responses, which are often marked by increased inflammatory cytokine release and the accumulation of inflammatory cells (Zeisel, Dhawan, and Baumert 2019). Our study aimed to assess these changes by analyzing fecal samples through 16S rDNA sequencing at the end of the experiment. The sequencing depth was validated through rarefaction and Shannon diversity curves (Figure S2), confirming a sufficient and representative acquisition of ASVs. We evaluated the alpha diversity using Chao1, Shannon, and Simpson indices, finding a notable decline in the Chao1 and Shannon indices and an elevation in the Simpson index among DSS‐treated mice, as shown in Figure 5A–C. However, CW treatment significantly reversed these trends, suggesting a restoration of microbiota diversity and richness. The principal coordinate analysis (PCoA) based on ASV‐level Bray‐Curtis distances (Figure 5D) demonstrated distinct segregation between the gut microbiota compositions of CW‐treated mice and those treated with DSS, with the CW group clustering more similarly to the control group. This analysis highlights the potential of CW to ameliorate disruptions in gut microbiota associated with inflammatory conditions.

At the phylum level, significant differences in gut microbiota composition among the groups were observed, as depicted in Figure 5E. The stacked bar chart showed that the samples were primarily composed of Bacteroidota, Firmicutes, and Proteobacteria at phylum level. Following CW treatment, there was a notable shift in these populations, characterized by a reduction in Bacteroidota and an increase in Firmicutes, as detailed in Figure 5F. Such adjustments resulted in a substantial rise in the Firmicutes/Bacteroidota (F/B) ratio, a metric previously linked to IBD severity (Ma et al. 2021), and depicted in Figure 5G. At the genus level, CW's impact was further demonstrated by a decrease in *Bacteroides*, *Alloprevotella*, and *Prevotellaceae*_*UCG*‐*001* genera that were enriched following DSS treatment. Conversely, an augmentation in *Lactobacillus* abundance was observed (Figure 5H,I). These adjustments highlight CW's role in rebalancing the gut microbiota, suggesting its potential as a therapeutic agent for inflammatory gut disorders.

### 3.6 Correlation Between Host Parameters and Key Bacterial Groups

**FIGURE 6:**
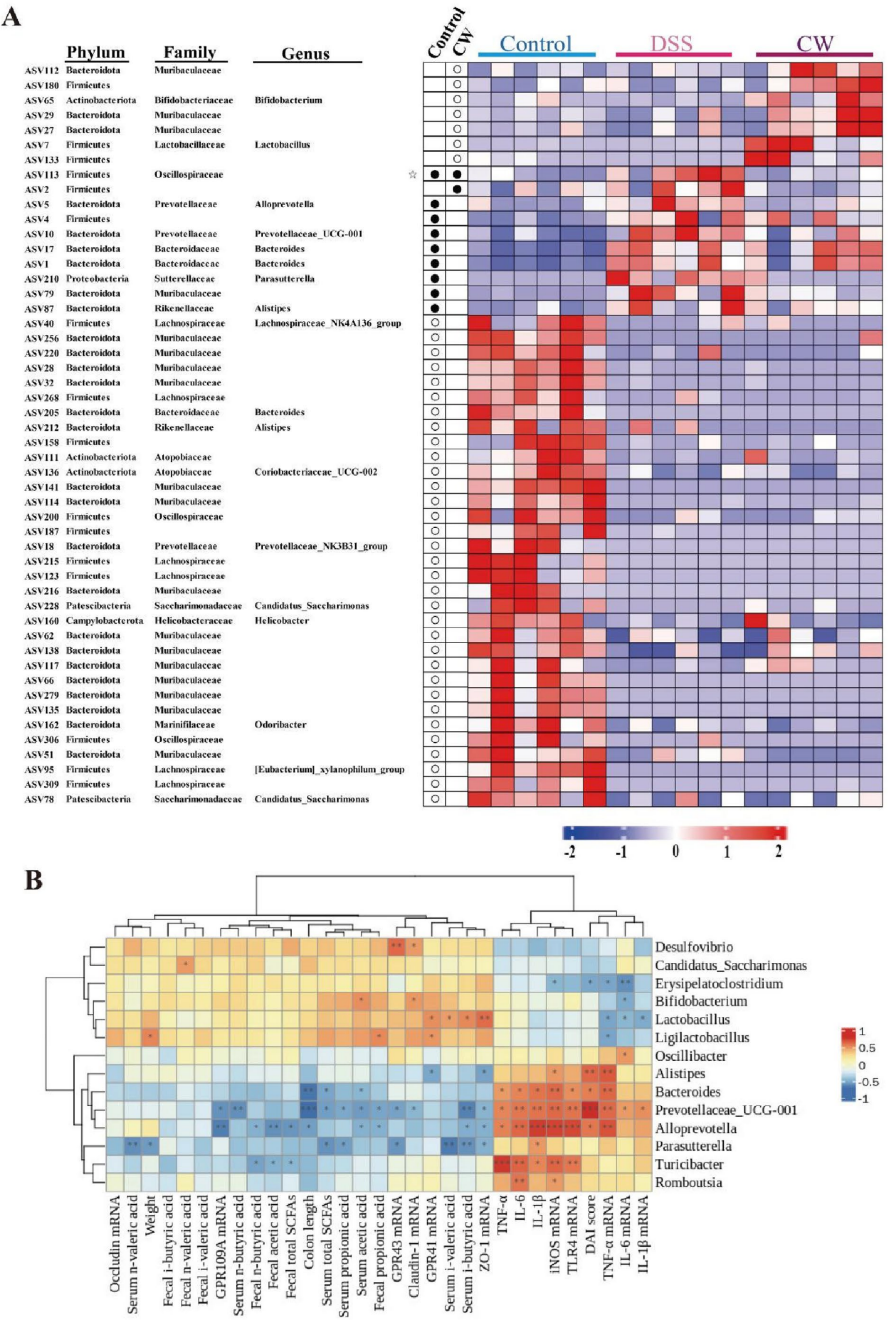
Influence of CW on gut microbiota composition at the ASV level and correlation analysis of colitis‐related biomarkers with key genera altered by CW treatment. The heatmap displays the relative abundance of 50 ASVs. Circles (○) and dots (●) represent ASVs that are lower or higher, respectively, in the Control and β‐Glucan groups compared to the DSS group, respectively. Stars (☆) indicate ASVs in the Control group altered by DSS treatment that were reversed after CW intervention. Red (blue) squares indicate positive (negative) Spearman correlation coefficient (R) values. Significant correlations are denoted by *p < 0.05, **p < 0.01, ***p < 0.001.

Considering the variable responses of individual ASVs within similar phyla or genera to dietary interventions, a detailed ASV‐level analysis was performed to delineate microbial populations pivotal in IBD progression. Focusing on ASVs exceeding a 0.1% relative abundance, the study found that DSS treatment significantly altered forty‐four ASVs: increasing nine and decreasing thirty‐five (*p* < 0.05), as illustrated in Figure 6A. Conversely, CW treatment enhanced seven ASVs while reducing two ASVs. Spearman correlation analysis investigated the links between these microbiota changes and colitis improvement post‐CW treatment, showing that beneficial microbes such as *Lactobacillus* and *Bifidobacterium* inversely correlated with inflammation markers (Figure 6B). These microbes also positively correlated with both SCFAs levels in serum and colon and the expression of their receptors. In contrast, detrimental bacteria like *Parasutterella*, *Alloprevotella*, and *Bacteroides* directly associated with severe colitis symptoms, including reduced colon length and elevated DAI scores. This data underscores the intricate connections among diet, gut microbiota composition, and inflammatory responses in IBD, suggesting these microbial dynamics as potential therapeutic targets.

## 4 Discussion

Mounting evidence underscores the complex pathogenesis of UC, which is closely linked to genetic predispositions, intestinal barrier dysfunction, imbalanced inflammatory responses, and microbial dysbiosis. In light of these factors, the development of effective new therapeutic agents is crucial. *C. auriculatum* Royle ex Wight contains various bioactive constituents, including polyphenols and alkaloids, which have shown promising anti‐inflammatory and antioxidant properties (Chai et al. 2018; Ding et al. 2019). The DSS‐induced UC model, which closely mimics the clinical and pathological features of human UC, such as weight loss, diarrhea, bloody stools, and colonic inflammation, is widely used to study disease mechanisms and evaluate new treatments (Wirtz et al. 2017). This study is the first to confirm the therapeutic effects of CW in a DSS‐induced colitis mouse model, significantly alleviating symptoms like weight loss, diarrhea, and bloody stools, and ameliorating colonic shortening, inflammatory cell infiltration, crypt damage, epithelial injury, and intestinal barrier dysfunction. These results underscore CW's promising role as a new treatment option for IBD. Inflammatory responses and intestinal barrier dysfunction are central to the pathology of IBD, serving as critical targets for understanding disease mechanisms and developing preventive and therapeutic strategies. However, the dose–response studies were not involved in the present work, which should be our next work.

Intestinal epithelial cells, crucial for forming a barrier between the human body and external elements, depend on tight junctions (such as Occludin, ZO‐1, and Claudin‐1) and transmembrane proteins (Beumer and Clevers 2021; Ma et al. 2022). These components are vital for regulating cell function, maintaining intestinal equilibrium, and controlling permeability (Zeisel, Dhawan, and Baumert 2019). Additionally, Muc‐2 glycoproteins secreted by goblet cells in the gut help maintain mucosal integrity by preventing direct contact between intestinal bacteria and the host mucosa (Gustafsson and Johansson 2022). Interestingly, the histopathological analysis in this work revealed that supplementation with CW effectively ameliorates the histological damage caused by DSS, including crypt hyperplasia, goblet cell depletion, submucosal edema, and dense inflammatory cell infiltration, thus preserving intestinal integrity. These results underscore the potential of CW as an innovative and effective treatment for IBD. Abnormal changes in the intestinal barrier can trigger aggressive immune responses in the gut, culminating in the extensive release of pro‐inflammatory cytokines such as IL‐1β, IL‐6, and TNF‐α, thereby exacerbating colonic injury (Bergemalm et al. 2021). The study in this work demonstrated that supplementation with CW prevents the excessive formation of TNF‐α, IL‐1β, and IL‐6 in the serum of mice with DSS‐induced colitis. Research on the anti‐colitic effects of CW explored its impact on gene expression related to the inflammatory response in the colon. The study found that CW notably reduced the mRNA levels of pro‐inflammatory markers including TNF‐α, IL‐1β, IL‐6, Toll‐like receptor 4 (TLR4), and iNOS, while it enhanced the expression of the anti‐inflammatory cytokine IL‐10 in colitis.

Studies in both IBD patients and mouse models have shown a rise in intestinal mucosal macrophages, which, through the NF‐κB pathway, significantly increase the production of pro‐inflammatory cytokines including TNF‐α, IL‐6, IL‐1β, and NO (Guan 2019). This promotes the progression of colitis, enhances inflammatory responses, disrupts tissue equilibrium, damages epithelial cells, and sustains a vicious cycle that exacerbates inflammation. Excessive production of IL‐6 may directly injure epithelial cells and trigger apoptosis, disrupting intestinal barrier function, increasing the permeability to pathogens and harmful substances, and exacerbating the inflammatory response (Katz, Zsiros, and Kiss 2019). High levels of IL‐6 can also inhibit the expression of IL‐10, weakening its anti‐inflammatory effects and leading to further spread and exacerbation of inflammation. Overexpressed TLR4 enhances its ligand binding, such as LPS, triggering pathways that escalate the release of pro‐inflammatory cytokines including TNF‐α, IL‐6, and IL‐1β, thus intensifying inflammation (Di Lorenzo et al. 2022). Elevated colonic iNOS gene expression increases nitric oxide levels, leading to oxidative stress, inflammatory cell infiltration, epithelial cell damage, and the production of pro‐inflammatory cytokines, thus exacerbating the development and severity of colitis (Kolios, Valatas, and Ward 2004). IL‐10, an essential anti‐inflammatory cytokine, is vital for immune homeostasis; its deficiency may result in severe inflammatory bowel diseases (Glocker et al. 2011). It promotes tissue protection and regeneration via several pathways, including reducing the production of pro‐inflammatory cytokines and enhancing the functionality of regulatory T cells (Saraiva, Vieira, and O'Garra 2019). Our research findings indicate that CW markedly diminishes the levels of pro‐inflammatory cytokines such as IL‐1β, IL‐6, and TNF‐α, while also safeguarding intestinal barrier integrity in a DSS‐induced model.

SCFAs, essential metabolites synthesized by gut microbiota, are key in maintaining intestinal balance and reducing colonic inflammation. SCFAs levels are often markedly reduced in colitis conditions (He et al. 2024). Research shows that CW supplementation significantly increases serum and fecal levels of acetic, propionic, butyric, and valeric acids. Butyric acid, crucial for the energy supply of intestinal mucosal cells, also enhances tight junction protein expression, thus decreasing intestinal permeability and preventing bacterial translocation (Fagundes et al. 2024). Additionally, valeric acid supplementation has been shown to attenuate DSS‐induced colitis in mice by inhibiting macrophage activity. Moreover, valeric acid has demonstrated effectiveness in reducing colitis in mouse models through macrophage inhibition (M. Liu et al. 2024). SCFAs exert their biological effects primarily by activating GPCRs on the surface of epithelial cells, which influence various downstream signaling pathways. For instance, GPR43‐deficient mice demonstrate exacerbated inflammation in colitis models, underscoring the anti‐inflammatory role of this receptor (Smith et al. 2013). Additionally, activating GPCR109A with SCFAs boosts anti‐inflammatory factor production, stimulates regulatory T‐cell activity, and fosters the development of IL‐10‐producing T cells, crucial for colitis prevention (Singh et al. 2014). Thus, the findings in this work suggested that CW supplementation may modulate the intestinal epithelial structure in DSS‐induced murine colitis through GPCR activation, positioning CW as a promising therapeutic option for managing colonic inflammation.

Dysbiosis of the gut microbiota is intricately linked to the pathogenesis of IBD, where imbalances in the intestinal microbiome can initiate or exacerbate inflammation (Franzosa et al. 2019). In this work, supplementation with CM not only enhances gut microbial diversity but also alters the overall microbiota structure to restore ecological balance. Firmicutes, a major component of the microbiota, enhance their resistance to inflammation through products of cell wall polysaccharide synthase, regulated by IL‐34 (Jordan et al. 2023). Clinical observations have noted that a rise in Bacteroidetes and a decline in Firmicutes correlate with increased disease severity in IBD patients (Y. Ma et al. 2021). In this research, CW treatment resulted in an elevation of Firmicutes levels and a reduction in Proteobacteria, with a consequent improvement in the F/B ratio, signaling a decrease in inflammation. At the genus level, CW treatment resulted in decreased relative abundances of *Bacteroides*, *Alloprevotella*, and *Prevotellaceae_UCG‐001*, alongside an increase in *Lactobacillus*. Specifically, strains like Enterotoxigenic *Bacteroides fragilis* , which are implicated in promoting intestinal inflammation, showed decreased levels (Chung et al. 2018). *Alloprevotella* is notably enriched in tumors overexpressing IL23A and IL1RN genes (Ge et al. 2020), while *Prevotellaceae_UCG‐001* is significantly more abundant in colorectal cancer mice (Ibrahim et al. 2019). Moreover, lactic acid bacteria (LAB), beneficial Gram‐positive microbes, not only produce acetic acid but also collaborate with butyrate producers to bolster butyrate levels, crucial for regulating immune functions within the gut and alleviating inflammation (Ren, Faas, and de Vos 2020). A specific instance involves *Lactobacillus reuteri* , which mitigates inflammation by influencing the Wnt/β‐catenin signaling pathway (Wu et al. 2020). Consequently, our results indicate that CW supplementation significantly enhances colitis management in mice by modulating the gut microbiota, thereby affirming its viability as a therapeutic option for IBD.

In conclusion, the defense mechanism of CW against DSS‐induced colitis is illustrated in Figure S3, which summarizes our findings. CW demonstrates considerable protective attributes against DSS‐induced colitis by altering the gut microbiota. This alteration enhances SCFAs production, bolsters intestinal barrier integrity, and diminishes epithelial permeability, collectively mitigating colitis symptoms. These findings suggest that CW can serve as an effective microbiota‐based strategy for both the prevention and management of IBD. Nonetheless, further exploration is warranted to identify the specific bioactive components and underlying mechanisms, such as NF‐κB and MAPK, through which CW influences the gut microbiota.

## 5 Conclusion

The results indicate that CW provides protective effects against colitis induced by DSS, demonstrated through significant improvements in clinical symptoms such as weight recovery, increased colon length, and decreased diarrhea and rectal bleeding. CW supplementation inhibits the high levels of inflammation induced by DSS, suppressing excessive inflammatory mediators (serum TNF‐α, IL‐1β, and IL‐6), downregulating the overexpressed mRNA levels of TNF‐α, IL‐1β, IL‐6, TLR4, and iNOS, while upregulating IL‐10, thereby alleviating colitis. Additionally, CW enhances gut barrier integrity and mitigates histological damage induced by DSS. It promotes goblet cell differentiation, increasing MUC2 production, and elevates the expression of tight junction proteins such as ZO‐1, occludin, and claudin‐1. Furthermore, CW promotes SCFAs enrichment, activates GPCRs (GPR43, GPR41, and GPR109A), and modulates the gut microbiota by enriching *Lactobacillus* and reducing *Bacteroides*, *Alloprevotella*, and *Prevotellaceae_UCG‐001*, thereby alleviating colitis and restoring intestinal barrier function.

## Author Contributions

**Sichen Li:** investigation (equal), methodology (equal), writing – original draft (equal). **Yuning Sun:** data curation (equal), formal analysis (equal), investigation (equal). **Huihui Peng:** investigation (equal). **Ruiqiang You:** data curation (equal). **Fuqing Bai:** data curation (equal), formal analysis (equal), software (equal). **Dan Chen:** writing – review and editing (equal). **Mohamed Abdin:** validation (equal), writing – review and editing (equal). **Chuanyi Peng:** conceptualization (equal), writing – review and editing (equal). **Xiang Li:** conceptualization (equal), funding acquisition (equal), resources (equal), writing – review and editing (equal). **Huimei Cai:** conceptualization (equal), funding acquisition (equal), project administration (equal), supervision (equal), writing – review and editing (equal). **Guijie Chen:** conceptualization (equal), funding acquisition (equal), methodology (equal), project administration (equal), supervision (equal), writing – review and editing (equal).

## Ethics Statement

This study was approved by the Animal Ethics Committee of Anhui Agricultural University (Approval No. AHAU2023028).

## Conflicts of Interest

The authors declare no conflicts of interest.

## Supporting information


Data S1.

## Data Availability

The data that support the findings of this study are available on request from the corresponding author.
