# Supplementary material for: Chemical Composition of Cynanchum auriculatum Royle Ex Wight and Its Potential Role in Ameliorating Colitis
Source: Food Sci Nutr. 2025 Jan 19;13(1):e4764. doi: 10.1002/fsn3.4764 (PMC11742642; doi:10.1002/fsn3.4764)
Supplement: Supplementary file 1 — Data S1. [file FSN3-13-e4764-s001.docx]

**Chemical Composition of *Cynanchum auriculatum* Royle ex Wight and Its Potential Role in Ameliorating Colitis**

Sichen Li^a^^,b^^,1^, Yuning Sun^a,b,1^, Huihui Peng^a,b^, Ruiqiang You^c^, Fuqing Bai^a,b^, Dan Chen^d^, Mohamed Abdin^e^, Chuanyi Peng^a,b^, Xiang Li^c,^*, Huimei Cai^a,b^^,^*^[[1]](#footnote-0)^ , Guijie Chen^a,b,^*

^a^ State Key Laboratory of Tea Plant Biology and Utilization, School of Tea & Food Science and Technology, Anhui Agricultural University, Hefei 230036, Anhui, PR China

^b^ Joint Research Center for Food Nutrition and Health of IHM, Anhui Agricultural University, Hefei 230036, Anhui, PR China

^c^ School of Marine and Biological Engineering, Yancheng Teachers’ University, Yancheng 224007, China

^d^ College of Food Science and Engineering, Yangzhou University, Yangzhou 225127, Jiangsu, China

^e^ Agricultural Research Center, Food Technology Research Institute, Giza 12611, Eg

**Table S1. Composition of daily diet in animal**

| Product# | Percentage |
| --- | --- |
| Protein | 22.9% |
| Carbohydrate | 66.0% |
| Fat | 11.1% |
| Total | 100.0% |
| kcal/kg | 3886 |

**Table S2. Disease activity index (DAI) scoring criteria**

| Score | Weight loss | Stool consistency | rectal bleeding |
| --- | --- | --- | --- |
| 0 | <1% | Normal | Normal |
| 1 | 1-5% | Between | Between |
| 2 | 5-10% | Loose stools | Slight bleeding |
| 3 | 10-20% | Between | Between |
| 4 | >20% | Watery diarrhea | Gross bleeding |

**Table S3. Histological colitis scoring criteria**

| Score | Standard |
| --- | --- |
| 0 | Normal |
| 1 | Mild inflammation and edema are observed in the mucosal layer, accompanied by the disappearance of the lower one-third of the crypts |
| 2 | Moderate inflammation is evident in the mucosal layer, concomitant with the loss of the lower two-thirds of the crypts at the base |
| 3 | Moderate inflammation is present in the mucosal layer, with the complete absence of crypts, yet the epithelial layer remains intact |
| 4 | Moderate inflammation is evident in the mucosal layer, submucosa, and muscular layer, with the concurrent loss of crypts and epithelial layers |

**Table S4. Primer sequences for quantitative qRT-PCR**

| **Genes** | **Foreward** | **Reverse** |
| --- | --- | --- |
| GADPH | AGGTCGGTGTGAACGGATTTG | TGTAGACCATGTAGTTGAGGTCA |
| IL-6 | TGAACAACGATGATGCACTTG | CTGAAGGACTCTGGCTTTGTC |
| IL-1β | TGCCACCTTTTGACAGTGATG | ATGTGCTGCTGCGAGATTTG |
| TNF-α | TCTGTCTACTGAACTTCGGGGTG | ACTTGGTGGTTTGCTACGACG |
| iNOS | GAGACAGGGAAGTCTGAAGCAC | CCAGCAGTAGTTGCTCCTCTTC |
| TLR4 | GCCATCATTATGAGTGCCAATT | AGGGATAAGAACGCTGAGAATT |
| IL-10 | AGTACAGCCGGGAAGACAAT | TCTAGGAGCATGTGGCTCTG |
| ZO-1 | CTGGTGAAGTCTCGGAAAAATG | CATCTCTTGCTGCCAAACTATC |
| Claudin-1 | ACTGGGTCAGGGAATATCCA | TCAGCAGCAGCCATGTACTC |
| Occludin | TGCTTCATCGCTTCCTTAGTAA | GGGTTCACTCCCATTATGTACA |
| GPR41 | CGACTAGAGATGGCTGTGGT | AGAAGATGAGCAGTGTGGCT |
| GPR43 | AATCAGAAGACAGAAAAGGAGCTG | TCTGGGGTCATTCTCCTTGG |
| GPR109A | TCCAAGTCTCCAAAGGTGGT | TGTTTCTCTCCAGCACTGAGTT |

**Table S5. Chemical constituents of CW.**

| No. | tR  min | Chemical  formula | Calculated  m/z | Observe  m/z | Error  ppm | MS/MS | Identificaton |
| --- | --- | --- | --- | --- | --- | --- | --- |
| 1 | 0.52 | C_6_H_14_N_4_O_2_ | 173.1042 | 173.1044[M-H]- | -1.16 | 59.0135, 71.0141, 131.0832, 154.0614 | L-Arginine* |
| 2 | 0.71 | C_6_H_8_O_7_ | 191.0209 | 191.0199[M-H]- | 5.24 | 59.0135, 87.0095, 143.0344, 161.0468 | Citric Acid* |
| 3 | 6.72 | C_48_H_82_O_20_ | 1023.5435 | 1023.5476[M-H]- | -4.01 | 146.9675, 211.8817, 316.7390 | Yesanchinoside B |
| 4 | 10.18 | C_42_H_66_O_16_ | 885.4559 | 885.4583[M-H]- | -2.71 | 126.9057, 146.9650, 211.8847, 316.7427, 469.1769 | Esculentoside A |
| 5 | 10.49 | C_18_H_34_O_5_ | 329.2337 | 329.2335[M-H]- | 0.61 | 146.9675, 171.1015, 211.8817 | Tianshic acid* |
| 6 | 10.65 | C_29_H_36_O_15_ | 341.1104 | 341.1089[M-H]- | 4.40 | 59.0135, 179.0561, 191.0209 | Isoacteoside* |
| 7 | 11.25 | C_48_H_78_O_18_ | 1001.5374 | 1001.5329[M-H]- | 4.49 | 146.9675, 197.96749, 211.8817, 316.7427, 919.5047, 955.5295 | Soyasaponin Bb* |
| 8 | 11.53 | C_53_H_86_O_21_ | 1117.58 | 1117.5942[M-H]- | -12.71 | 146.9675, 197.9649, 211.8817, 1053.5697 | 3-O-α-L-rhamnopyranosyl-(1→2)-α-L-arabinopyranosyl-28-O-β-D-glucopyranosyl-(1→6)-β-D-glucopyranosyl oleanolate |
| 9 | 12.18 | C_54_H_88_O_21_ | 1131.5851 | 1131.5953[M-H]- | -9.01 | 112.9863, 129.9758, 146.9675, 279.2344, 595.2921, 1085.5753 | Buddleoside* |
| 10 | 12.98 | C_52_H_84_O_21_ | 1043.5436 | 1043.5431[M-H]- | 0.48 | 146.9675, 197.9649, 881.0613, 997.5402, 937.5247 | Yuzhizioside IV |
| 11 | 12.26 | C_16_H_32_O_2_ | 274.2785 | 274.2739[M+H]+ | 16.77 | 149.0262, 184.0772, 230.2524, 256.2658 | Palmitic acid* |
| 12 | 13.74 | C_23_H_28_O_6_ | 339.2019 | 339.2001[M-H]- | 5.31 | 126.9057, 144.9252, 183.0128, 248.9600 | Songoroside A* |
| 13 | 14.62 | C_31_H_48_O_7_ | 311.1694 | 311.1689[M+H]+ | 1.61 | 129.9758, 146.9675, 155.9416, 183.0152, 579.2875 | Phytolaccagenin |
| 14 | 15.61 | C_18_H_32_O_3_ | 295.2322 | 295.2282[M-H]- | 13.55 | 129.9758, 146.9675, 277.2177 | Coronaric acid* |
| 15 | 15.89 | C_54_H_88_O_21_ | 595.2921 | 595.2899[M-H]- | 3.70 | 146.9675, 152.9976, 241.0125, 279.2344, 315.0528, 415.2309 | Prosapogenin 5 |
| 16 | 15.99 | C_54_H_86_O_23_ | 1109.5729 | 1109.5617[M+H]+ | 10.09 | 145.0879, 184.0772, 345.2113, 511.2735, 821.4112 | Lablaboside A* |
| 17 | 16.8 | C_32_H_44_O_9_ | 571.2911 | 571.2894[M-H]- | 2.98 | 127.0766, 146.9675, 255.2326 | Gandoeric acid H* |
| 18 | 17.78 | C_35_H_56_O_7_ | 309.2065 | 309.2035[M+H]+ | 9.70 | 141.1147, 149.0262, 184.0744, 201.0494 | Raddeanoside R0* |
| 19 | 17.99 | C_31_H_48_O_7_ | 325.1861 | 325.1847[M-H]- | 4.31 | 129.9758, 146.9675, 183.0152, 255.2326 | Phytolaccagenin |
| 20 | 20.58 | C_20_H_34_O_2_ | 313.279 | 313.2735[M+H]+ | 17.56 | 133.0882, 141.1174, 171.1409 | Ethyl linolenate |
| 21 | 22.29 | C_22_H_29_NO_2_ | 398.2345 | 398.2337[M-H]- | 2.01 | 116.9276, 129.9758, 144,9252, 146.9675, 183.0125, | Lobelanidine* |
| 22 | 22.68 | C_28_H_36_O_10_ | 532.2328 | 532.2253[M+H]+ | 14.09 | 141.1147, 149.0262, 358.3142, 4406.3316, 56.4619 | Nomilinic acid* |
| 23 | 23.19 | C_21_H_42_O_4_ | 381.302 | 381.2971[M+H]+ | 12.85 | 96.0866, 141.1172, 149.0262, 184.0072, 341.3136, 358.3142 | Glycerol monostearate* |
| 24 | 24.87 | C_55_H_90_O_25_ | 1151.6075 | 1151.5929[M+H]+ | 12.68 | 145.0879, 184.0772, 239.1306, 423.2439 | Tribulosin |
| 25 | 27.44 | C_73_H_118_O_37_ | 792.8629 | 792.8604[M-H]- | 3.15 | 112.9863, 126.9057, 144.9252, 180.9751, 248.9632, 316.9506, 384.9429 | Asterbatanoside D |
| 26 | 29.25 | C_17_H_22_O_3_ | 281.1776 | 281.1723[M+H]+ | 18.85 | 101.0606, 111.0453, 145.0903, 184.0772, 267.1693 | 3β-Acetoxy-atractylone |
| 27 | 29.9 | C_29_H_36_O_15_ | 624.206 | 624.1988[M+H]+ | 11.53 | 281.0541, 355.0754 | Isoacteoside* |
| 28 | 30.28 | C_37_H_58_O_12_ | 701.4166 | 701.4085[M+H]+ | 11.55 | 149.0462, 221.0886, 141.1147, 327.0833, 663.4631, 685.444 | Cimicifuga Dahurica C |
| 29 | 30.32 | C_31_H_48_O_7_ | 325.186 | 325.1849[M-H]- | 3.38 | 112.9863, 146.9675, 183.0152 | Phytolaccagenin |
| 30 | 32.11 | C_69_H_112_O_37_ | 773.3601 | 773.3536[M-H]- | 8.40 | 133.0882, 226.9572, 282.2837, 587.559, 647.4644 | Platycoside D* |
| 31 | 35.15 | C_16_H_25_NO_2_ | 141.1147 | 141.1135[M+H]+ | 8.50 | 90.9771, 95.0866, 124.0901 | Lycopodium Alkaloids* |
| 32 | 38.77 | C_73_H_118_O_37_ | 792.8629 | 792.8604[M-H]- | 3.15 | 112.9863, 180.9751, 248.9632, 316.9506, 384.9429, 452.928 | Asterbatanoside D |

* referring to identification by comparison with a references


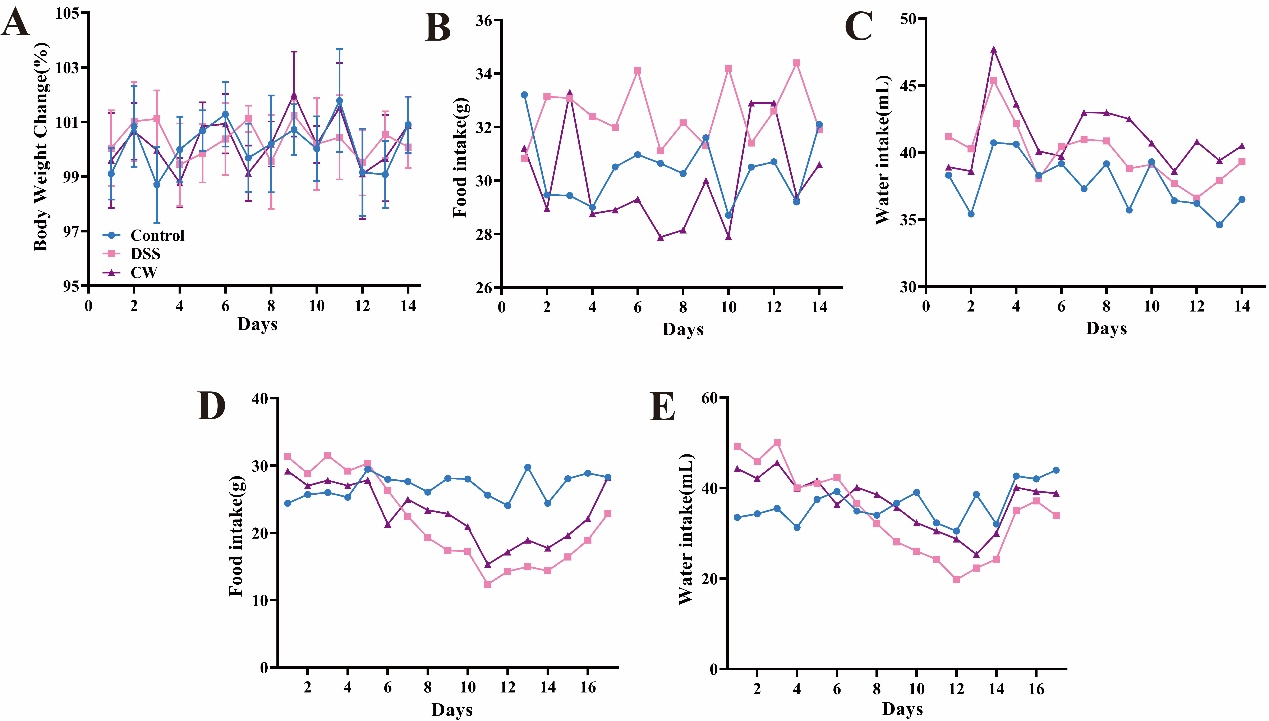


**Fig. S1. Pre-DSS Administration: Body Weight and Water Consumption Changes.** The pre-administration body weight percentage (A), food intake (B), and water consumption (C) of mice, as well as the changes in water (D) and food intake (E) post-DSS administration in mice.


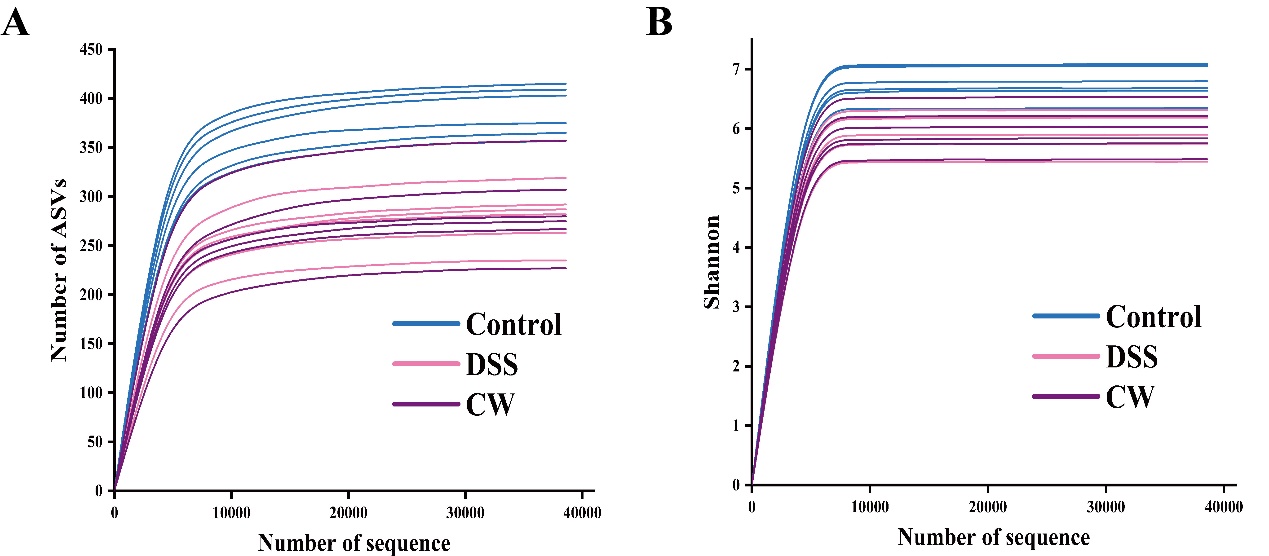


**Fig. S2.** Mouse stool samples (A) dilution curve and (B) Shannon index.


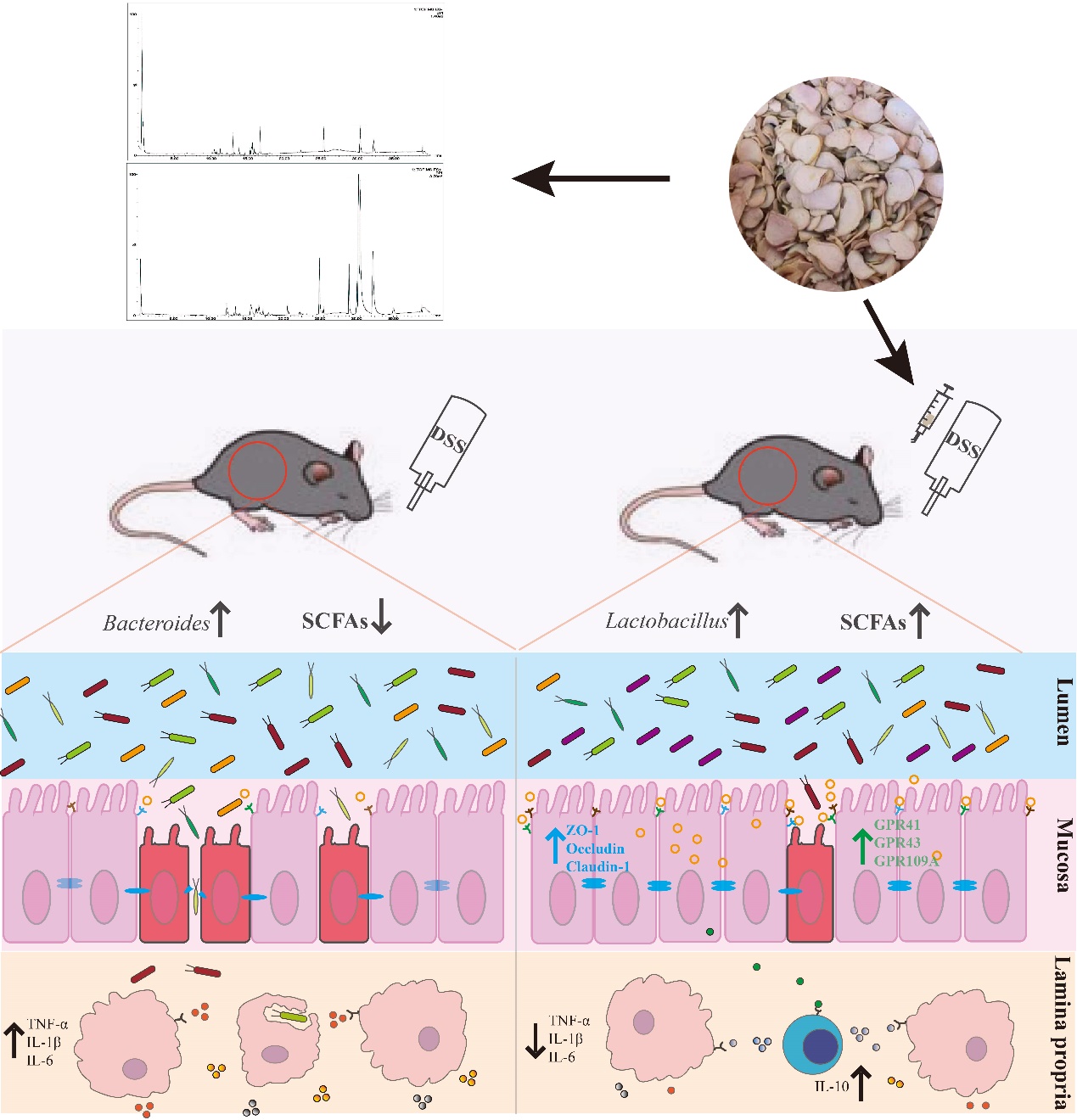


**Fig. S3.** Proposed mechanism of colitis attenuation by CW

1. * Corresponding author. E-mail: lix01@yctu.edu.cn (X. Li); chm@ahau.edu.cn (H. Cai); [guijie@ahau.edu.cn](mailto:guijie@ahau.edu.cn) (G. Chen)

   ^1^ These authors contributed equally to this work. [↑](#footnote-ref-0)
